# *In silico* and *in vitro* analysis of boAP3d1 protein interaction with bovine leukaemia virus gp51

**DOI:** 10.1371/journal.pone.0199397

**Published:** 2018-06-21

**Authors:** Adriana Patricia Corredor, Janneth González, Luis Alfredo Baquero, Hernando Curtidor, Nury Nathalia Olaya-Galán, Manuel Alfonso Patarroyo, María Fernanda Gutiérrez

**Affiliations:** 1 Virology Laboratory, Universidad Javeriana, Bogotá DC, Colombia; 2 Nutrition and Biochemistry Department, Science Faculty, Universidad Javeriana, Bogotá DC, Colombia; 3 Molecular Biology and Immunology Department, Fundación Instituto de Inmunología de Colombia (FIDIC), Bogotá DC, Colombia; 4 Basic Sciences Department, School of Medicine and Health Sciences, Universidad del Rosario, Bogotá DC, Colombia; 5 PhD Programme in Biomedical and Biological Sciences, Universidad del Rosario, Bogotá DC, Colombia; National Institutes of Health, UNITED STATES

## Abstract

The envelope glycoprotein 51 (gp51) is essential for bovine leukaemia virus (BLV) entry to bovine B-lymphocytes. Although the bovine adaptor protein 3 complex subunit delta-1 (boAP3D1) has been proposed as the potential receptor, the specific ligand-receptor interaction has not yet been completely defined and boAP3D1 receptor and gp51 3D structures have not been determined. This study was thus aimed at a functional annotation of boAP3D1 cellular adaptor protein and BLV gp51 and, proposing a reliable model for gp51-AP3D1 interaction using bioinformatics tools. The boAP3D1 receptor interaction patterns were calculated based on models of boAP3D1 receptor and gp51 complexes’ 3D structures, which were constructed using homology techniques and data-driven docking strategy. The results showed that the participation of 6 key amino acids (aa) on gp51 (Asn170, Trp127, His115, Ala97, Ser98 and Glu128) and 4 aa on AP3D1 (Lys925, Asp807, Asp695 and Arg800) was highly probable in the interaction between gp51 and BLVR domains. Three gp51 recombinant peptides were expressed and purified to validate these results: the complete domain (rgp51), the N-terminal portion (rNgp51) and the C-terminal fragment (rCgp51); and binding assays to Madin-Darby bovine kidney (MDBK) cells were then carried out with each recombinant. It was found that rNgp51 preferentially bound to MDBK cells, suggesting this domain’s functional role during invasion. The rNgp51-MDBK cell interaction was sensitive to trypsin (98% reduction) and chymotrypsin treatment (80% reduction). These results highlighted that the N-terminal portion of gp51 interacted *in vitro* with the AP3D1 receptor and provides a plausible *in silico* interaction model.

## Introduction

The bovine leukaemia virus (BLV) is a retrovirus from the same genus as the human T-cell leukaemia-lymphoma virus (HTLV) [1], displaying tropism mainly to cattle B-lymphocytes [2], where it has been associated with a slow infection similar to human acquired immunodeficiency syndrome (AIDS), known as enzootic bovine leukosis. This disease is characterised by having no evident symptomatology in 65% of infected animals, causing persistent lymphocytosis in 30% of them and leukaemia or lymphoma in 5% to 10% [3–5].

The pertinent literature has reported that this virus infects cells other than B-lymphocytes and even cells from species other than cattle [6–10]; it has also been reported recently that it is present in women’s mammary gland cells, suggesting the virus’ association with breast cancer [11,12]. The cellular protein candidate for viral receptor, allowing virus entry to these cells, must be studied to advance understanding of how BLV can infect cells other than B-lymphocytes, such as human epithelial cells and sheep T-lymphocytes.

The BLV envelope (Env) protein, comprising a 51 kDa molecular weight surface (SU) domain (called gp51), a transmembrane (TM) domain (known as gp30) and a cytoplasmatic (CP) domain, has been involved in virus binding to and penetration of cells [13,14].

Two studies have been published to date referring to the BLV cell receptor. Two receptor (BLVR)-related clones (BLVcp1 and BLVcp1/5’) were found in the first experimental approach, encoding a plasmatic membrane protein whose extracellular domain binds BLV gp51 and increased the susceptibility of cells to recombinant BLV infection [15,16]. A later study proposed that BLVR was related to the adaptor-related protein complex-3 (AP-3) which participates in intracellular protein transport [17]; the MDBK cell line was used for the experiments in both studies. There are currently 75 complete BLV genome sequences in GenBank; only two proteins, a capsid (CA) [18] and a transmembrane protein [19] have been resolved by crystallography. Using computational tools to understand the function of the proteins involved in binding is therefore an important step in resolving concerns about BLV biology.

Generally speaking, *in silico* approaches have been of key importance in assessing protein-protein interactions [20,21]; such methods were used here for identifying functionally important protein regions. Some *in vitro* approaches have been used regarding BLV to identify the cellular receptor [15–17]. The present study describes the functional annotation of BLV gp51 and boAP3D1 proteins and predicts their interaction (GenBank Accession No M35242.1. and No NP_776423). BLV Env and boAP3D1 protein tertiary structures were here modelled and analysed for identifying domains and binding sites and identify and functionally characterise infection pathway components which could lead to a better understanding of BLV pathogenesis and provide pharmacological targets.

Three gp51 recombinant proteins were constructed for determining their Madin-Darby bovine kidney (MDBK) cell binding capability, under the premise that AP3D1 is a cell membrane molecule present in these cells. This was done as a first validation of the *in silico* results which showed an interaction between gp51 and boAP3D1; these results were quite promising according to *in vitro* tests, opening the way forward for further studies aimed at clarifying the receptor involved in BLV infection and also solving gaps in tropism, pathogenesis and maybe identifying future vaccine targets.

## Materials and methods

### Computational analysis of primary structure

The BLV Env and AP3D1 proteins primary sequences were retrieved from GenBank (http://www.ncbi.nlm.nih.gov/) accession numbers M35242.1 (FLK-BLV isolate used as BLV reference strain), NP_776423.3 (AP3D1 bovine boAp3F1).ProtParam [22] was used for calculating protein physical-chemical properties, such as molecular weight, theoretical pI, aa composition, atomic composition, extinction coefficient, estimated half-life, instability index, aliphatic index and grand average of hydropathicity (GRAVY).

The ProtScale tool in the ExPASy server was used for boAP3D1 and Env protein aa scale representation (Kyte & Doolittle hydrophobicity scale) [23], having an aa scale defined by a numerical value assigned to each type of aa. The most frequently used scales are hydrophobicity or hydrophilicity scales and secondary structure conformational parameter scales; there are many other scales based on aa chemical and physical properties. The ProtScale tool provides 57 predefined scales entered from the literature [22].

### Functional annotation

BLV Env and AP3D1 conserved domains were analysed by sequence similarity search with close orthologous family members available in various protein databases using the web-tools CDD-BLAST [24], INTERPROSCAN [25,26] and COGs [27,28] for this purpose. PROSITE [29] was used for identifying patterns and profiles.

### Secondary structure prediction

The PDBSum [30] and CDD_BLAST servers [24] were used for computing and analysing protein sequence secondary structural features. The NSP server (https://npsa-prabi.ibcp.fr/cgi-bin/npsa_automat.pl?page=/NPSA/npsa_seccons.html) gave average results from the consensus of 5 algorithms using two basic methods: probability parameters determined by relative frequencies and Bayesian probabilities. GlobPlot tools [31] were used for identifying boAPd1 and gp51 intrinsically disordered proteins (IDPs).

### 3D structure prediction

The Env and boAP3D1 proteins’ 3D structure was predicted by using measures for each type of aa in local structural environments and defined in terms of solvent accessibility and protein secondary structure. Coat protein complex I (COPI) was used for Env; COPI is involved in traffic between the Golgi apparatus and the endoplasmic reticulum [32]. Several criteria were taken into account when selecting the template for modelling, such as crystal resolution, sequence similarity (% identity), conserved regions/domains and sequence coverage. Despite the 3D structure of the BLV Env transmembrane region being available, the main goal of the present study was to characterise the surface (SU) domain (gp51), since this is directly involved in the interaction with the cell receptor and thus, following the above-mentioned criteria, COPI turned out to be the best template choice.

The clathrin-associated AP2 adaptor complex was used for boAP3D1 as it plays roles in many vesicle trafficking pathways within cells [33]. COPI (PDB ID 5A1U) and AP2 adaptor complex (PDB ID 2VGL) crystal structures were the templates selected for obtaining the 3D structures of Env and boAP3D1, respectively. GROMOS96 force field (http://www.gromacs.org) [34] was used for quality and reliability assessment once the 3D model had been obtained and energy minimisation performed. Structural evaluation and stereochemical quality was evaluated.

### Molecular docking simulations

Scripps Research Institute (http://www.scripps.edu/mb/olson/doc/autodock) Autodock software (v4.2) (Autodock, Autogrid, Autotors, Copyright- 1991e2000) was used for Env protein and AP3D1 docking analysis. A searching grid extended over the selected target protein to delimitate the docking area was used to run Autodock. Polar hydrogens were added to ligand moieties, Kollman charges assigned and atomic solvation parameters added. Gasteiger polar hydrogen charges were assigned and nonpolar hydrogens were merged with the carbons; internal degrees of freedom and torsions were set. AP3D1 was docked with target protein, being this molecule considered a rigid body. Affinity maps for all atom types and an electrostatic map were computed (0.375 E grid spacing). The Lamarckian genetic algorithm selected in Autodock was used for the search.

### Refinement and complex validation

MacroModel (softwarehttps://www.schrodinger.com/macromodel) was used for screening docking solutions for energy minimisation to avoid steric overlaps and clashes. A 0.05 kJ/A° -mol was set as convergence criterion for gradient minimisation for protein–protein complex and docking performance quality test.

### Computing binding free energy

Distance-scaled, finite ideal-gas reference (DFIRE) state energy software [35] was used for assessing the complex’s (Env and AP3D1) binding free energy and estimating binding affinity. PyMOL (the PyMOL Molecular Graphics System, version 2.0 Schrödinger, LCC) was used for polar contact assessment.

### Mapping protein-protein interactions

PyMOL (the PyMOL Molecular Graphics System, version 2.0 Schrödinger, LCC) was used for visualising and mapping interactions between BLV Env and AP3D1 aa.

### Obtaining recombinant protein gp51

BLV DNA was extracted from a blood sample collected from a serologically positive bovine. For this purpose, a LymphoSep density gradient (MP Biomedicals) was used for obtaining peripheral blood mononuclear cells (PBMCs). A High Pure PCR Template Preparation kit (Roche) was then used for extracting total DNA for obtaining proviral DNA, following the manufacturer’s indications.

PCR amplification of the *gag* gene was used for confirming BLV presence in the sample, using previously reported primers [11]. The proviral DNA was then used as template in PCR reactions for which specific primers were designed for amplifying gp51 fragments from the FLK reference sequence deposited in the NCBI database (accession number M35242). Such regions were gp51 Nt aa 35–173 (Fwd 5' ATGAGATGCTCCCTGTCCCTAG 3' and Rev 5' TAAAGAAAAGGTGATCAGGGG 3'), gp51 Ct aa 173–301 (Fwd 5' ATGTTACATAAGATCCCTGATCCC and Rev 5' ACGTCTGACCCGGGTAGG 3') and the complete gp51 (aa 35–301), using the gp51 Nt forward and gp51 Ct reverse primers. A Wizard PCR Clean-Up System kit (Promega) was used for purifying PCR products; amplicon quality was then evaluated on 1.5% agarose gels. The purified products were ligated into pEXP5-CT/TOPO expression vector (Invitrogen) and each recombinant construct was used for transforming *E*. *coli* TOP-10 cells (Invitrogen). Several recombinant clones were grown for plasmid DNA extraction with an UltraClean mini plasmid prep purification kit (MO BIO Laboratories). Insert integrity and correct orientation were confirmed by Sanger sequencing (Macrogen, Seoul, South Korea). ClustalW NPS software [36] was used for determining similarity between FLK reference strain *gp51* gene sequences and that isolated from bovine sera.

Once the *gp51* sequence was confirmed, the pEXP5-gp51 (complete, NT and CT) recombinant plasmids were transformed in *E*. *coli* BL21-DE3 cells (Invitrogen), following the manufacturer’s recommendations. Once the cells had reached a 0.5 DO_600_, 1 mM IPTG (Sigma-Aldrich) was added to induce molecule expression for 4h at room temperature with constant shaking at 250 rpm. *E*. *coli* BL21-DE3 bacteria were recovered by spinning and the cell pellet was used for extracting recombinant proteins in denaturing conditions.

After verifying expression by Western blot, all recombinant proteins were purified from whole cell lysate supernatants by affinity chromatography using Ni^2+^-NTA resin (Qiagen). The mixture was left overnight at 4°C and then passed through a chromatography column; exhaustive dialysis was carried out twice to obtain the three recombinant proteins in a functional conformation. The first was carried out inside the column before elution, using decreasing concentrations of urea buffer (3, 1.5, 0.75, 0.37 M in PBS 1X adding 1mM reduced glutathione, 0.1 mM oxidised glutathione). The fractions obtained after elution were dialysed with PBS 1X pH 7.2 for 72 h at 4°C to eliminate remaining urea and enable proper recombinant refolding. This procedure has been described by Singh, S. *et al*., as being effective for obtaining a proper conformation and function for proteins expressed in *E*. *coli* and extracted as denatured protein [37]. We therefore think that the three fragments so obtained had the proper conformation and were functionally active; however, additional assays are required to confirm correct recombinant folding.

All the fractions collected were analysed by 12% SDS-PAGE and Western blot; those presenting just one band were dialysed in 1X PBS at pH 7.2. A BCA protein assay micro kit (Thermo scientific) was used for quantifying the proteins which were ultra-filtered and concentrated with Amicom Ultra-4 centrifugal filters (Merck Millipore).

Verifying protein expression involved separating purified recombinant proteins (rgp51, rNgp51 and rCgp51) (10 μg) by 12% SDS-PAGE and then transferring it to a nitrocellulose membrane and incubating with a peroxidase conjugated (1:4,500) monoclonal anti-histidine antibody (A7058, Sigma-Aldrich) recognising these recombinant proteins’ histidine tail. The membranes were revealed with a peroxidase substrate kit (Vector Laboratories), according to the manufacturer’s recommendations. The proteins’ molecular masses were determined by linear regression using the XL-OptiProtein (New England Biolabs) molecular mass marker as reference.

### Evaluating recombinant protein capability to bind to MDBK cells

MDBK (ATCC, #CCL-22 derived from bovine kidney) cells were cultured with Dulbecco’s modified Eagle’s medium (DMEM) (Sigma, D5523) containing L-glutamine and 1,000 mg/L glucose, supplemented with 3.7 gm/L sodium bicarbonate and 10% foetal calf serum.

The next step involved radiolabelling 15μg complete (rgp51) or N-terminal (rNgp51) or Carboxy-terminal (rCgp51) recombinant proteins with 4 μL Na^125^I (100 cpm/mL; ARC) and Iodination Beads (Pierce-Thermo Scientific), following the manufacturer’s instructions. Following 12 min of incubation, radiolabelled recombinant protein was separated by size-exclusion chromatography on a Sephadex G-25 column (Pharmacia). Each eluted fraction was then analysed by gamma counter (Packard Cobra II).

Binding assays involved 1.2×10^6^ MDKB cells being incubated with 150 and 300 nM concentrations of each radiolabelled recombinant protein at room temperature for 90 min in the absence (total binding) or presence (non-specific binding) of 13μM of the same unlabelled recombinant protein. The cells were spun through a 60:40 dioctyl phthalate -dibutyl phthalate cushion (1.015 g/ml density, 10,200 x *g* for 1.5 min) and a gamma counter (Packard Cobra II) was used for quantifying cell-associated radioactivity.

Each recombinant protein’s binding activity was also evaluated in a binding assay with enzyme-treated cells. Briefly, cells were independently treated with 1mg/mL trypsin (Sigma T-1005) or 1 mg/mL chymotrypsin (Sigma C-4129) for 60 min at 37°C. Following incubation, enzyme-treated cells were washed twice with HBS buffer and used in a typical binding assay. Untreated cells were used as positive binding control.

## Results

### Computational analysis of primary structure

**Fig 1:**
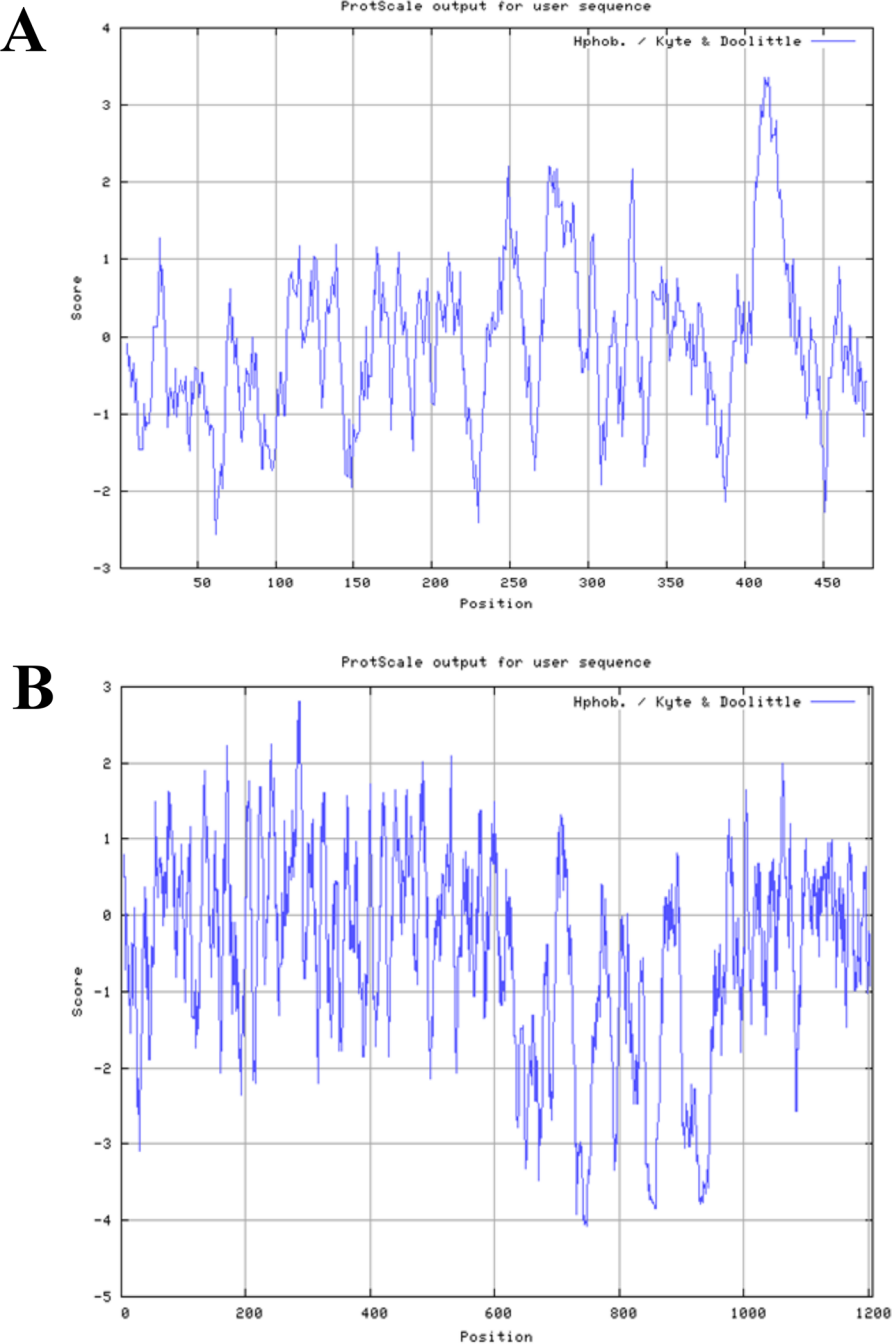
**Kyte & Doolittle hydrophobicity for BLV Env (A) and boAP3D1 (B) proteins.** Despite differences regarding the amount of aa in both proteins, their physicochemical characteristics were comparable.

Table 1 gives boAP3D1, BLV Env and recombinant proteins’ physicochemical properties. The Env protein consists of 515 residues, 33 of which form part of the signal peptide, 36 are positively charged and 33 negatively charged. The boAP3D1 protein has 1,207 residues; 173 of them are positively charged and 179 negatively charged. Grand average of hydropathy (GRAVY) was also calculated, thereby determining that the proteins were hydrophilic as the resulting value was negative, favouring protein solubility in water. Fig 1 shows Kyte & Doolittle hydrophobicity for boAP3D1 and gp51 proteins. The physicochemical properties of the three recombinant proteins (rgp51, rNgp51 and rCgp51) are also described.

**Table 1:** boAP3D1, BLV Env and recombinant proteins physicochemical properties.

Property	Value				
	boAP3D1	BLV Env	rgp51	rNgp51	rCgp51
Amount of aa	1,207	515	268	140	129
Molecular weight KDa	136	54	30	16	14
Theoretical pI	6.65	8.19	7.74	7.10	8.06
Total amount of negatively charged residues (Asp+Glu)	179	33	19	12	7
Total amount of positively charged residues (Arg+Lys)	173	36	20	12	8
Ext. coefficient M−1 cm−1	75,845	120,735	74,410	39,795	34,615
Instability index (all are unstable protein)	49.79	49.17	51.57	45.22	58.02
Aliphatic index	88.86	95.93	74.57	65.32	86.98
Grand average of hydropathicity (GRAVY)	-0.490	-0.092	-0.346	-0.458	-0.194

**Fig 2:**
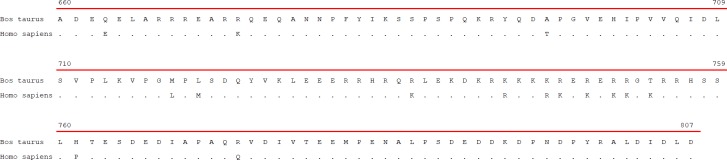
ClustalOmega sequence alignment of boAP3D1 and huAP3D1. “-” represents identical aa, the red line represents the BLVR domain.

Aligning bovine and human AP3D1 protein sequences with ClustalOmega software (https://www.ebi.ac.uk/Tools/msa/clustalo/) revealed 15 differences in the BLVR domain whilst no differences between human and bovine peptide were found in the adaptin domain. The red line in Fig 2 represents the BLVR domain.

### Functional annotation

**Fig 3:**
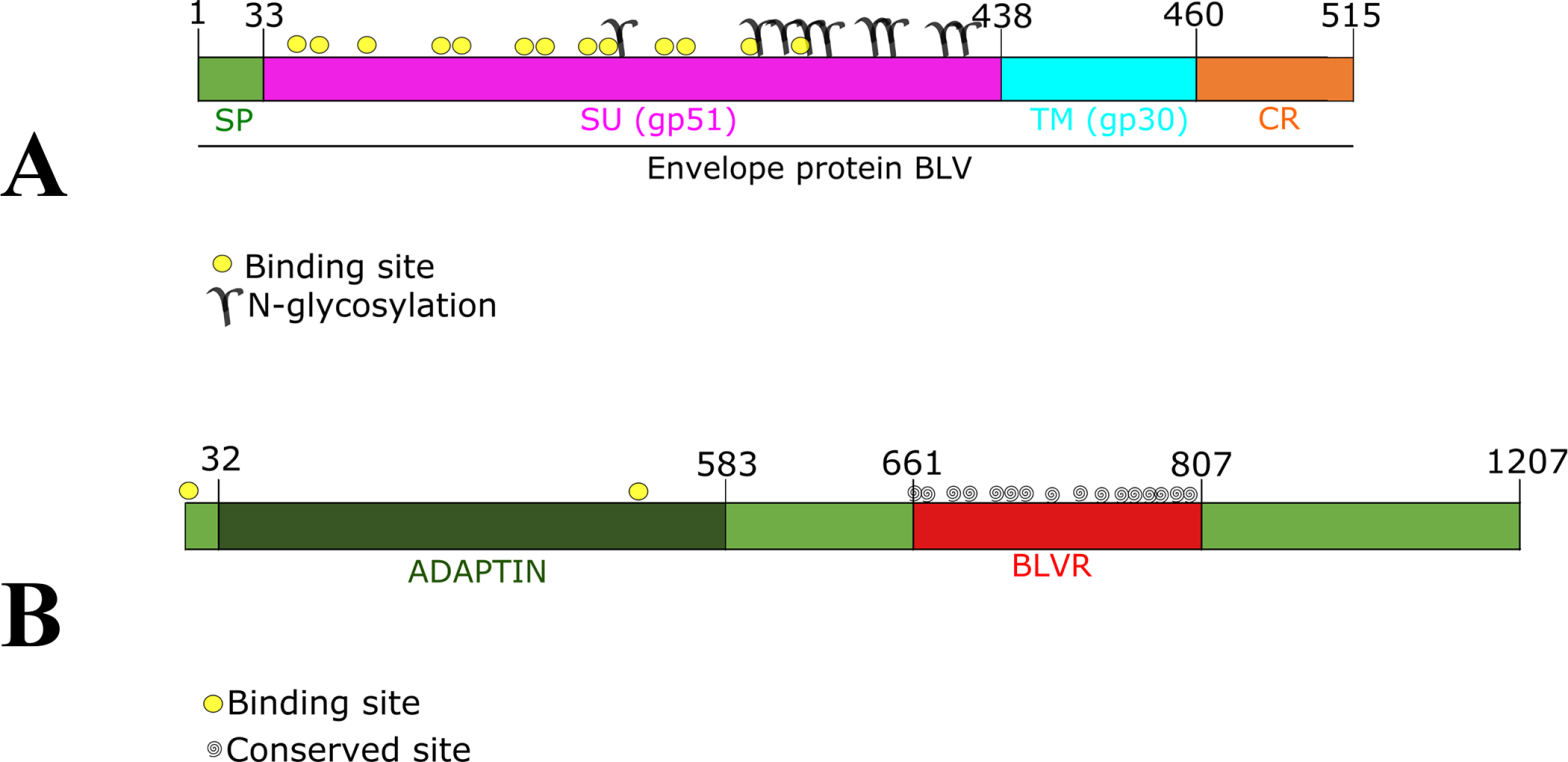
Schematic representation of Env BLV and boAP3D1 proteins. 3A. BLV Env protein. Signal peptide 1 to 34, SU (gp51) 34 to 438, TM (gp30) 439 to 460 and cytoplasmic region 461 to 515. Binding sites are shown by yellow circles (48, 50, 77, 98, 99, 112, 113, 120, 122, 135, 136, 187, 292 and 298), N-glycosylation sites (129, 203, 230, 251, 256, 271, 287, 351 and 398). 3B. boAP3D1 protein has 1,207 aa with two domains: one from aa 32 to 583 for adaptin (dark green) and a second (BLVR) from aa 661 to 807 (red). AP3D1 has 2 binding sites in positions 1 and 474 (yellow dots) and 16 conserved sites in the BLVR domain (D661, E662, S686, S688, K726, E728, E729, K739, D767, E779, E783, A785, L786, S788, D797 and A801).

The BLV Env protein is 515 aa long; it is located on cell membrane and has three domains. The extracellularly-located initial domain consists of a 33 aa-long signal peptide followed by an extracellular region from aa 34 to 438 (SU or gp51). The intermediate portion is a transmembrane region from aa 439 to 460 (TM or gp30) and the final portion is a cytoplasmic region from aa 461 to 515. Fourteen aa (48, 50, 77, 98, 99, 112, 113, 120, 122, 135, 136, 186, 187, 292 and 298) which can be considered binding sites are found all along the SU. Fig 3A shows the 14 binding aa in yellow and the 9 glycosylation sites (aa 129, 203, 230, 251, 256, 271, 287, 351 and 398).

The 1,207 aa-long boAP3D1 protein has two domains: the adaptin domain (residues 32–583) and the BLVR domain (residues 661–807) which is a disorganised region, like others all along the protein. Fig 3B shows the scheme for this protein, having 2 binding sites in positions 1 and 474 and 16 conserved sites in the BLVR domain (D661, E662, S686, S688, L726, E728, E729, D739, D767, E779, E783, E785, E786, S788, D797 and A801).

### Secondary structure prediction

The BLV envelop protein’s secondary structure consists of 37 α-helices, 41 helix-helix interaction regions, 77 β-turns and 22 γ-turns. The boAP3D1 protein has 3 β-sheets, 3 β-hairpins, 2 β-bulges, 7 strands, 4 α-helices, 2 helix-helix interaction regions, 15 β-turns and 2 γ-turns. The boAP3D1 protein has 10 disordered regions in positions 3–8, 39–64, 80–99, 139–157, 181–189, 200–208, 223–228, 255–264, 443–456 and 472–482 and a globular domain between aa 158 to 442.

The AP3D1 intrinsically disordered protein (IDPs) is distributed in 12 regions in positions 120–128, 190–198, 632–637, 680, 695, 714–718, 784–802, 873–878, 943–947, 964–970, 1033–1051 and 1182–1190 and four globular domains between aa 1–679, 696–783, 803–1032 and 1052–1207.

Analysis performed with the PROSITE [29] database predicted that the boAP3D1 gp51 binding domain would be located between aa 660 and 807 (Fig 3B, shown in red).

### 3D models predicted for env and boAP3d1

**Fig 4:**
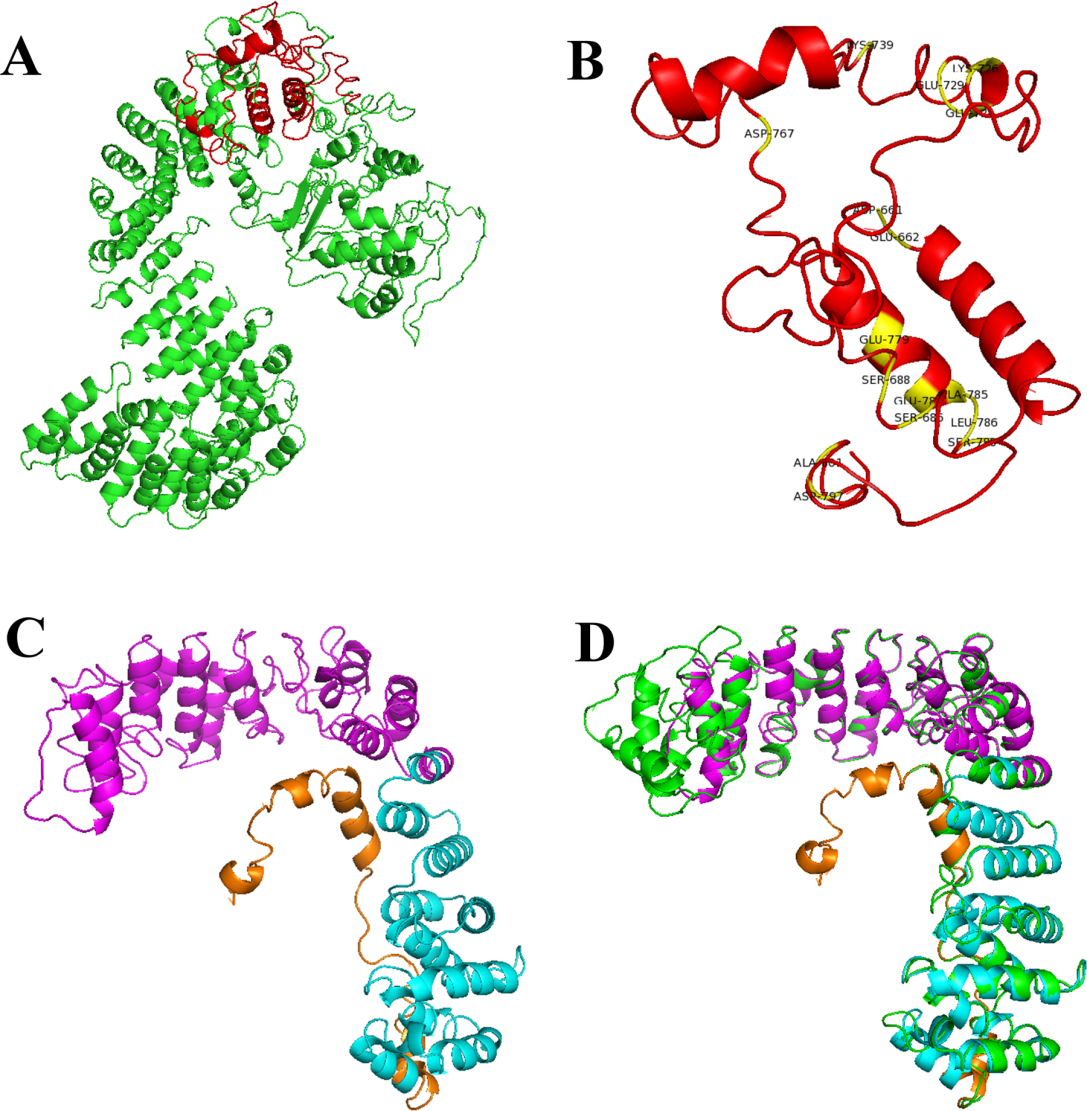
Modelling boAP3D1 and Env proteins. 4A. boAP3D1 structure is coloured green, and in red the BLVR domain. 4B. The BLVR domain (red) with conserved sites highlighted in yellow (D661, E662, S686, S688, K726, E728, E729, K739, D767, E779, E783, A785, L786, S788, D797 and A801). 4C. BLV Env protein structure predicted by I-TASSER, gp51 (SU) is shown in magenta, gp30 (TM) in cyan and cytoplasmic domain in orange. 4D. The COPI coat triad structure (5A1U in green) overlaps with our BLV Env protein model.

Fig 4 shows the predicted model for both proteins; Fig 4A shows boAP3D1 structure where green represents the binding domain (BLVr) and Fig 4B shows this domain in red and conserved areas in yellow (i.e. aa D661, E662, S686, S688, K726, E728, E729, k739, D767, E779, E783, A785, L786, S788, D797 and A801). Fig 4C shows Env 3D structure; magenta represents the gp51 domain (SU), cyan gp30 (TM), orange the cytoplasmic domain and yellow conserved residues A15, Y17, R44, R65, R66, E78, P79, D87, F89, Q102, G103, Q153, L154, S259 and R265.

The predicted model for the Env protein by Ramachandran plot [38] showed that 68.4% of the residues were located in the most favoured regions, 24.6% residues in additional allowed regions, 2.6% residues in generously allowed regions and 4.3% residues in disallowed regions. DFIRE was -607.58, reflecting the model’s quality; lower energy would have indicated that the model was closer to the native conformation. A perfect structure overlap with 5A1U was shown in this study, suggesting the model’s high quality (Fig 4D).

### Molecular docking simulations, complex refinement and validation and binding free energy computation

**Fig 5:**
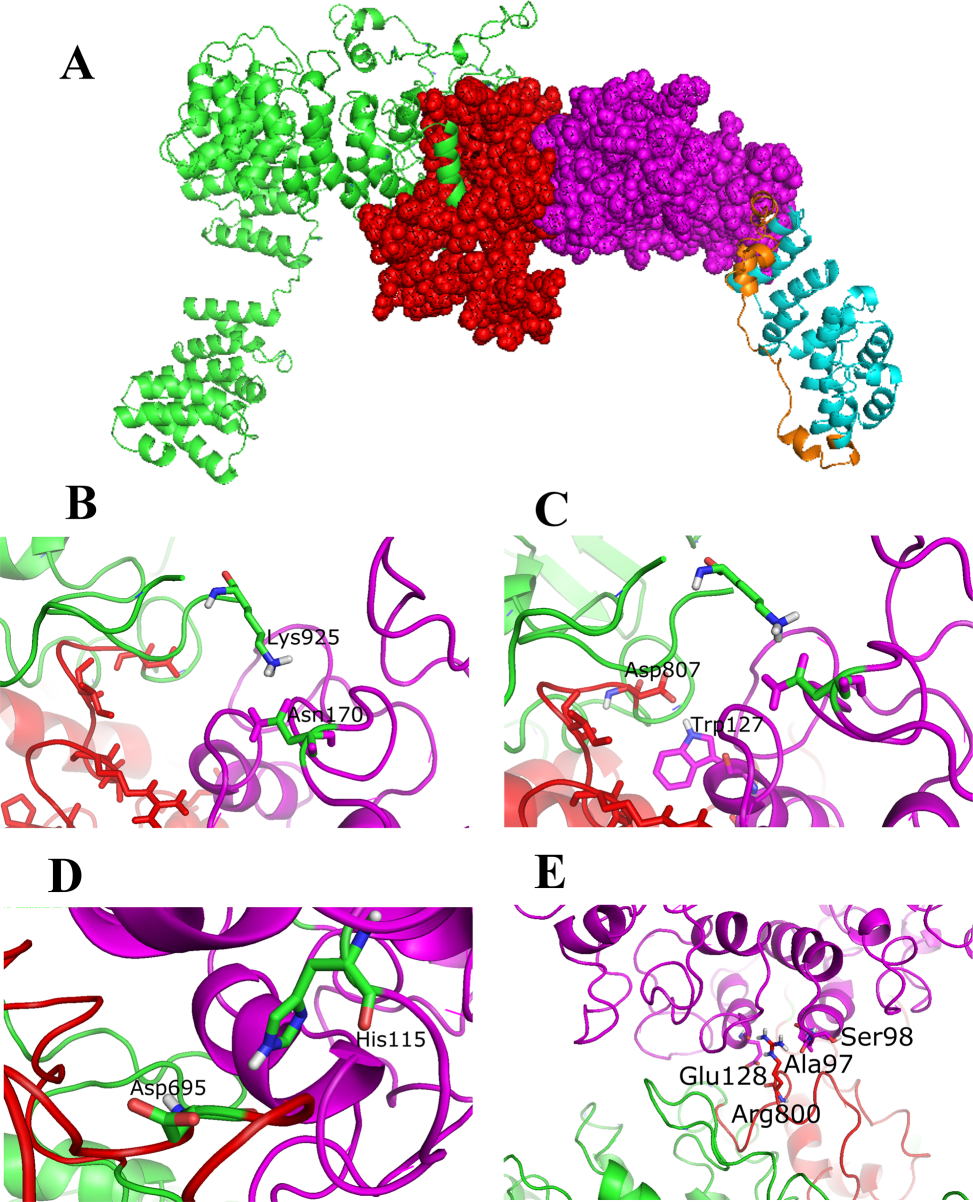
boAP3D1 and gp51 docking. 5A. Overview. 5B. Asn170 (gp51) and Lys925 (boAP3D1). 5C. Trp127 (gp51) and Asp807 (boAP3D1). 5D. His115 (gp51) and Asp695 (boAP3D1), hydrogen bonds, non-bonded contacts. 5E. Ala97, Ser98, Glu128 (gp51) and Arg800 (boAP3D1), hydrogen bonds, non-bonded contacts and salt bridges.

PyMOL was used for visualising molecular docking between gp51 and boAP3D1 (Fig 5). The part of the boAP3D1 protein making direct contact with gp51 was the BLVR domain (Fig 5A, red). The BLV-gp51 residues interacted with the proposed receptor (boAP3D1). The interface areas (A^2^) were 1,495 for boAP3D1 and 1,443 for gp51. This model obtained a score of -131.7, 14 cluster size, 23.6 RSMD for the overall lowest-energy structure, -50.4 Van der Waals energy, -470.4 electrostatic energy, -27.7 solvation energy restraints, 405.2 violation energy, 2,120.2 buried surface area and -1.9 Z-Score.

### Protein-protein interaction prediction

Possible interactions in the complex regarding Asn170 and Lys925 (Fig 5B), Trp127 and Asp807 (Fig 5C), His115 and Asp695 (Fig 5D), Ala97, Ser98, Glu128 and Arg800 participation in boAP3D1 (BLVR domain) and Env (gp51 domain) were analysed (Fig 5E). Table 2 gives the values calculated for binding free energy between these aa, i.e. 4 salt bridge interactions, 14 hydrogen bonds and 167 no direct contact points.

**Table 2:** Binding free energy results for gp51 and boAP3D1 interaction.

gp51	AP3D1	Hydrogen bonded	Non-bonded contacts	Salt bridges
		Distance (A)		
Asn170	Lys925	2.8	3.69	-
Trp127	Asp807	2.89	3.77	-
His115	Asp695	2.90	3.46	-
Ala97	Arg800	2.76	3.30	-
Ser98		3.20	3.20	-
Glu128		2.97	2.97	2.97

An additional *in silico* test showed the effect of mutating the predicted interaction residues in the binding energies between boAP3D1 and gp51. Two experimentally testable predictions were thus made; mutations leading to drastic chemical shifts were made in gp51 amino acids predicted as being crucial in binding to boAP3D1 (His115, Glu128 and Asn170 were replaced by Ala) and ClustalOmega software (https://www.ebi.ac.uk/Tools/msa/clustalo/) was used for multiple sequence alignment to assess whether the predicted interaction residues were conserved amongst the 10 BLV gp51 genotypes reported so far.

**Fig 6:**
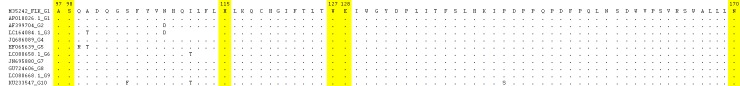
ClustalOmega sequence alignment of different BLV gp51 sequences (genotypes 1 to 10). “-” represents identical aa. The six key amino acids in the interaction are shown in yellow (Ala97, Ser98, His115, Trp127, Glu128 and Asn170).

As a result, binding energies’ native spectrum changed (-12.9 to -9.8 ΔG (Kcal mol^-1^), with a difference of 3.1). This could have led to decreased stability and loss of interaction. On the other hand, key amino acids in the interaction found here were highly conserved amongst different BLV genotypes (highlighted in yellow in Fig 6).

### Evaluating recombinant proteins’ MDBK cell binding capability

gp51 contains 9 N-glycosylation sites, most of them towards the C-terminus. Although the most appropriate expression system for expressing a glycoprotein would have been a eukaryotic one, previous studies by Rizzo *et al*., published in 2016 [39] have shown that gp51 interaction with its receptor was mediated by specific domains but sugars did not play any role in such interaction, as syncytia formation with maintenance of BLV particle infectivity remained when N-glycosylation sites of gp51 were mutated. A prokaryotic system was thus used here, taking its advantages into account, in terms of cost and ease of use.

**Fig 7:**
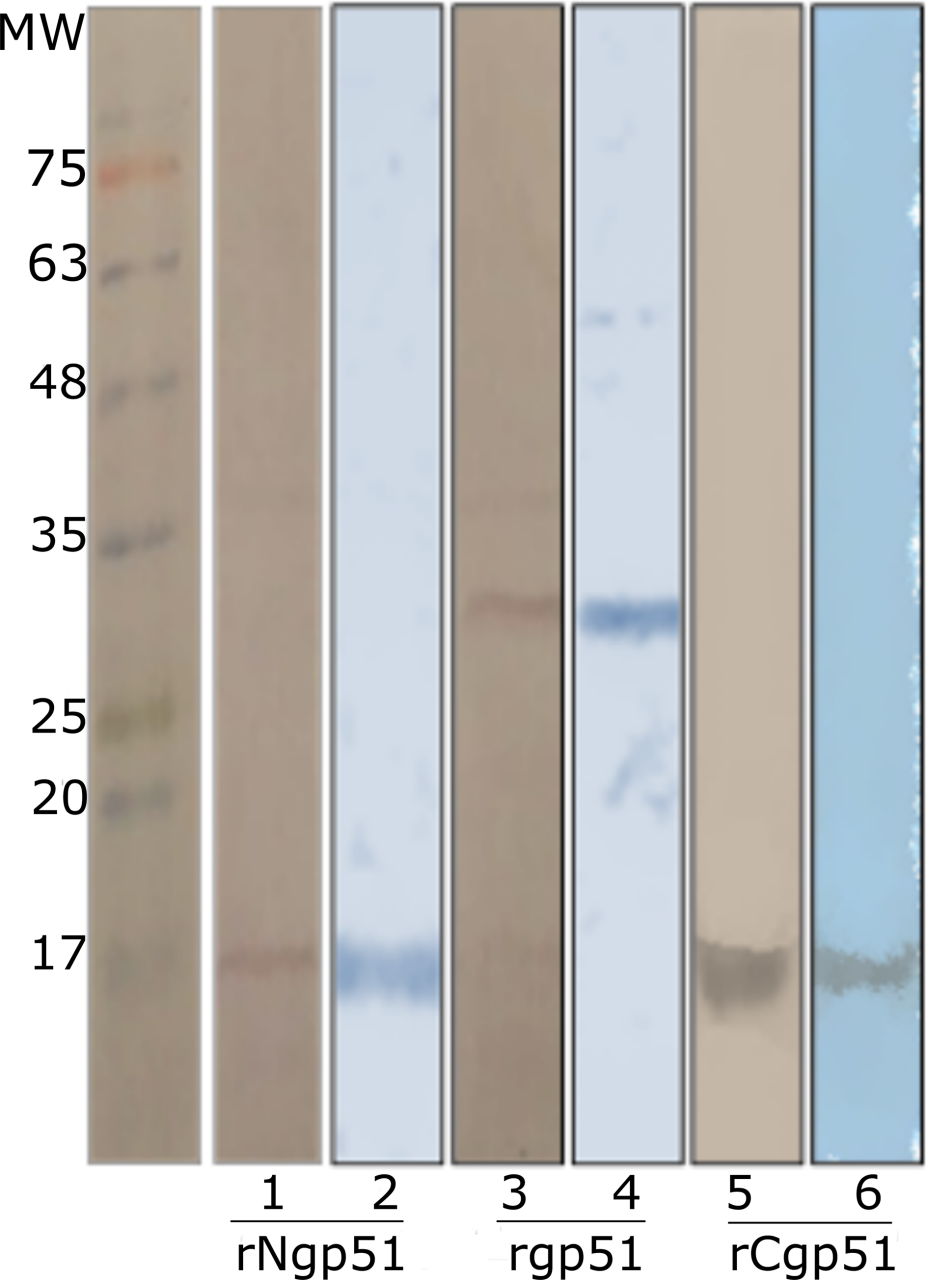
gp51 recombinant fragment purification. Lanes 1, 3 and 5 show Western blot detection with an anti-his monoclonal antibody. Lanes 2, 4 and 6 show Coomassie blue stained purified recombinant proteins. The proteins’ molecular weight marker is indicated in the first lane (molecular masses for the three recombinants agreed with expected ones: 30, 16 and 14 kDa for rgp51, rNgp51 and rCgp51, respectively).

Fig 7 shows gp51 recombinant fragments’ purification. Western blot detection is shown with Coomassie blue stained anti-his tag monoclonal antibody, i.e. the expected weight and purity of each recombinant fragment.

**Fig 8:**
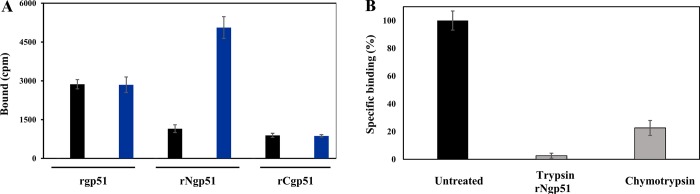
gp51 recombinant protein binding assays. 8A. rgp51 MDBK cell binding. Low rgp51 protein concentration (black bars) gave greater MDBK cell binding; however, rgp51 and rCgp51 binding did not change when protein concentration was duplicated (blue bars), whilst rNgp51 increased. 8B. MDBK cell interaction with rNgp51. The black bars represent enzymatically-treated MDBK and rNgp51 binding to the proposed receptor (AP3D1), followed by trypsin and chymotrypsin treatment, resulting in reduced in rNgp51 (98%) and MDBK binding (80%).

Binding assays determined rgp51 protein binding to MDBK cells. It was found that rgp51 had higher MDBK cell binding at low protein concentration (black bars) (Fig 8A); however, when protein concentration was increased (blue bars), a clear preferential binding was observed for rNgp51, whilst rgp51 and rCgp51 interaction remained the same.

Taking into account that binding experiments showed N-terminal fragment (rNgp51) preferential binding to MDBK cells, further experiments only involved this recombinant; furthermore, *in silico* analysis had also shown that the key interacting residues (Ala97, Ser98, His115, Trp127, Glu128 and Asn170) all lay within such fragment (aa 35 to 173). The gp51 N-terminal domain binding to MDBK cells was thus concentration dependent, suggesting this domain’s functional role during invasion; rNgp51-MDBK cell interaction was sensitive to trypsin and chymotrypsin treatment, binding becoming reduced by 98% and 80%, respectively (Fig 8B).

## Discussion

Bovine enzootic leukosis (LBE) is an infection affecting cattle and seems to have been restricted to such species to date [2]; however, there is evidence of it passing to humans, and some authors have proposed a relationship with breast cancer in women [11,12,40,41]. The possibility of a zoonosis is supported by the virus appearing in milk and meat from cows proving seropositive for BLV which could be acting as vectors of viral transmission to humans [42]. The possibility of being a zoonotic virus and its impact on public health mean that studies are needed in the search for the viral receptor enabling infection in two genetically distinct species (i.e. cattle and humans).

Studies *in silico* (like that described in this article) are necessary as a basis for initiating experimental studies verifying gp51 interaction with AP3D1, complementing existing information about BLV’s cellular receptor to achieve infection.

According to Env primary and secondary sequence characterisation, the primary sequence is extremely conserved amongst different BLV strains (0.0 e-value) (data not shown). High sequence identity with human (88%), mouse (88%), sheep (99%) and goat (99%) sequences was found for boAP3D1 (0.0 e-value), suggesting a common ancestor regarding this protein.

PSI-BLAST predicted 88% identity between bovine and human protein for AP3D1 and 99% with sheep and goat protein (data not shown). Aligning bovine and human AP3D1 protein sequences with ClustalOmega software (https://www.ebi.ac.uk/Tools/msa/clustalo/) revealed 15 differences in the BLVR domain whilst no differences between human and bovine peptide were found in the adaptin domain. The red line in Fig 2 represents the BLVR domain. (Fig 2); however, docking analysis (Fig 5) showed that they were not involved in the interaction with gp51 (Fig 2, in red). Most substitutions were conservative and just one of them was drastic (between Ala and Thr); such residues have different chemical properties, but such position was not predicted to be relevant for the interaction.

Properties supposed from the proteins’ tentative functions (i.e. homology, main domains, structural similarities and physical-chemical characterisation inferred by the predictions) provide useful information and should be verified experimentally.

The boAP3D1 envelope protein binding region and BLV entry to target cells requires virus-encoded glycoprotein gp51 to interact with cell receptors to facilitate virus entry. It has been proposed that gp51 plays an important role in virus entry to target cells during the viral cycle [43]. The protein’s most exposed aa should interact with specific receptors on target cells. A previous study reported that a protein similar to AP3D1 enabled BLV fusion and entry, but no specific boAP3D1 binding regions were established [15–17]. Regarding BLV and boAP3D1 docking assay results, it was found here that the AP3D1 interacting region with gp51 (located from aa 660–803) was the same region called the BLVR domain in previous studies (Figs 3 and 5).

Furthermore, considering BLV Env proteins, it has been shown that a region mediating interaction with a tentative receptor is located in gp51 or SU domain between aa 83–158. Some receptor binding domains (RBDs), zinc ion linker and binding and glycosylation sites have previously been described in this specific region [13] (Fig 3A). These regions could thus be crucial for a first interaction between host and viral proteins thereby mediating viral attachment. Fig 5 shows the most relevant interactions between both proteins’ specific aa as follows: Asn170-Lys925 (5B), Trp127-Asp807 (5C), His115-Asp695 (5D). Some interactions were mediated by hydrogen bonds, non-bonded contacts, as in Ala97, Ser98, Glu128 and Arg800 (5E), and hydrogen bonds, non-bonded contacts and salt bridges. Table 2 gives the distances between interactions. Interestingly, when alanine replacement led to drastic chemical shifts in some gp51 amino acids predicted as crucial in binding to boAP3D1 (His115, Glu128 and Asn170), the binding energy became drastically changed, thereby supporting the importance of such residues in the interaction.

The present study has suggested a possible hypothesis for viral entry to cells being AP3D1 protein-mediated as there is relevant similarity between this protein in different species and it could thus be said that this virus may have a binding pattern which is not species-specific at all and is using a ubiquitous receptor for achieving viral entry [8,44].

As other studies have proposed that the virus could enter other cells, it leads to a novel possibility involving a co-receptor being involved in cell infection [45,46] as AP3D1 can be found in a wide range of cells, so an additional molecule would seem to be required for BLV to acquire target cell and/or host specificity.

More recent studies have found the virus in other cell systems, particularly in the brains of cattle suffering neurological syndromes, suggesting different target cells being ultimately susceptible to viral infection and suggesting that this virus might be associated with other conditions than classically studied pathology [7]. These studies have presented a different perspective in which cattle are not really the only host which might be affected, as well as only lymphocyte cells being susceptible to infection by the virus.

However, it is clear that AP3D1 is not B-lymphocyte-specific, as shown by the BioGPS search (http://biogps.org) fed with experimental data accounting for this protein’s presence in different cell types (including human kidney cells), and the *in vitro* experiments in this work showing recombinant gp51 binding (NT fraction and complete protein) to MDBK cells (Fig 8A), whose binding was inhibited by enzymatic treatment (Fig 8B). The above is supported by these cells containing AP3D1 as, according to the present work, this protein’s similarity seems to locate it on the membrane (https://www.ncbi.nlm.nih.gov/pmc/articles/PMC2238969/); the Locate (http://locate.imb.uq.edu.au/) and QuickGO databases (https://www.ebi.ac.uk/QuickGO/) predicted the same.

This study adopted an in-depth bioinformatics and *in vitro* approach, searching for an explanation for gp51 interaction and its receptor. This is the first *in silico* approach to understanding BLV interaction with its host, further confirmed experimentally.

## Conclusions

Knowledge concerning the boAP3D1 cellular adopter protein and BLV gp51 has been expanded through these results. Molecular modelling and protein docking methods were useful for obtaining boAP3D1-receptor and gp51 complex 3D structures, showing these proteins’ interaction in detail. These models suggested the receptor-ligand interactions which could be occurring in BLV infection, leading to viral binding and fusion regarding viral entry. Further *in vitro* analyses confirmed that both the N-terminal region and the whole gp51 bound to MDBK cells, being the binding of the former region the strongest. Future studies aimed at assessing the potential use of the interactions here described for developing drugs or vaccines are thus recommended.
